# Evaluation of Integrative Medicine in Residency-Psychiatry Curriculum

**DOI:** 10.1007/s40596-024-02090-7

**Published:** 2024-11-15

**Authors:** Amelia Villagomez, Rajan Dunne, Audrey J. Brooks, Mei-Kuang Chen, Mari Ricker, Sophia Kang, Noshene Ranjbar

**Affiliations:** 1 University of Texas Southwestern Medical Center, Dallas, TX USA; 2 University of Arizona, Tucson, AZ USA; 3 MercyOne, Des Moines, IA USA

**Keywords:** Integrative medicine, Psychiatry, Patient-centered care, Education, Medical

## Abstract

**Objective:**

This study describes knowledge change, self-assessed confidence in providing integrative medicine approach, and respondents’ perspective on value and feasibility for the Integrative Medicine in Residency-Psychiatry (IMR-Psychiatry) curriculum, a 100-h elective offered during psychiatry training.

**Methods:**

Residents/fellows completed pre-post Medical Knowledge tests and self-assessment instruments tracking self-rated confidence. Participants were also interviewed for feedback about their experience using a semi-structured design after completion of the program.

**Results:**

Thirty-five of 37 (94.6%) participants completed all elective requirements. Items on the self-assessment instrument with the greatest positive pre-post change (*p* < 0.001) included ability to identify authoritative sources about botanicals (Cohen *d* = 2.15), effectively respond to patients’ questions regarding the use of herbs/supplements (Cohen *d* = 2.67), and interpret labels on nutraceuticals (Cohen *d* = 2.28). Mean score on the Medical Knowledge test (*n* = 30) increased from 64.7% correct at pretest to 81.6% at posttest. Responses tracking self-assessed confidence in providing an IM approach to address 18 common medical and psychiatric conditions all increased significantly pre to post (*p* < 0.001). Qualitative interviews provided important insights into challenges and recommendations for improvement, and all participants highly recommended the curriculum and found it feasible to complete during training.

**Conclusion:**

The IMR-Psychiatry curriculum provides trainees skills that promote comprehensive person-centered care; as a tool to optimize patient care and safety while enhancing physician well-being, wider-spread incorporation of the curriculum into psychiatry residency programs would be beneficial.

As defined by the Academic Consortium for Integrative Medicine and Health (ACIMH), integrative medicine “reaffirms the importance of the relationship between practitioner and patient, focuses on the whole person, is informed by evidence, and makes use of all appropriate therapeutic and lifestyle approaches, healthcare professionals and disciplines to achieve optimal health and healing” [1]. Integrative medicine blends conventional medicine with complementary and alternative medicine (CAM) practices that have been scientifically validated as safe and effective. CAM includes a diverse group of treatment approaches that are not presently included in conventional medical practice. Although many modalities within CAM meet these qualifications, others do not. While many patients use CAM for mental health concerns, they are often hesitant to discuss this with providers, and providers often do not inquire nor find themselves well equipped to advise around safe and optimal use of CAM [2, 3].

Since 1999, ACIMH has been supporting education, research, and policy collaborations among academic health centers with programs in integrative medicine. In addition, board certification in integrative medicine is available through the American Board of Integrative Medicine [4], under the American Board of Physician Specialties. As awareness of integrative medicine and related research expands, interest in incorporating this knowledge into medical education is growing with many medical schools now including multiple aspects of integrative medicine into curricula.

The Integrative Medicine in Residency (IMR) curriculum is a key example of an integrative medicine approach integrated into residency/fellowship trainings nationally and internationally. IMR was developed by the University of Arizona Andrew Weil Center for Integrative Medicine (AWCIM) in 2008 for family medicine residency training and has been evaluated and found to be feasible and effective at improving medical knowledge [5, 6]. IMR has been adapted for internal medicine, pediatrics [7], preventive medicine, obstetrics and gynecology, and emergency medicine.

To incorporate integrative medicine training into psychiatry residency, it was necessary to consider the unique needs of psychiatry training programs. A national needs assessment of psychiatry residency faculty and program directors identified a need for an integrative medicine curriculum in psychiatry residency. In addition, respondents stated that a standardized self-paced online curriculum supplemented by experiential learning was the preferred approach for implementation [8].

Recognizing interest and need, the University of Arizona College of Medicine-Tucson (UACOMT) Department of Psychiatry collaborated with AWCIM to develop and evaluate Integrative Medicine in Residency-Psychiatry (IMR-Psychiatry), a rigorous elective curriculum for psychiatric trainees, adapted from the original IMR, and co-designed by board-certified psychiatry faculty [9]. The goals of IMR-Psychiatry are to improve knowledge of evidence-based integrative medicine in psychiatry, enhance confidence in providing an integrative medicine approach to psychiatric care, and enhance resident self-awareness and well-being in the process. These goals were built on the premise that a psychiatrist’s ability to attune to another’s suffering is significantly influenced by the physician’s own self-awareness and self-care skills and that this type of training is often lacking in psychiatry residency [10]. The curriculum supports residents in meeting several Accreditation Council for Graduate Medical Education Milestones and Common Program Requirements for Psychiatry in the areas of patient care, medical knowledge (MK), practice-based learning and improvement, professionalism, and well-being [11].

IMR-Psychiatry is composed of an asynchronous interactive online component (~ 100 h) and an in-person experiential component. The online curriculum contains evidence-based modules on various integrative medicine modalities (e.g., mind–body medicine, nutraceuticals, and nutrition) [9]. The experiential sessions included a 10-week mind–body skills group [12], in addition to didactics and case discussions.

The mind–body skills component of the elective was designed to promote resident wellness, skill building, and sharing in small groups. In this group, residents learned the biological underpinnings of mind–body medicine and participated in group experiential sessions on topics such as mindfulness, biofeedback, and guided imagery [12]. Case discussions and didactics were utilized to teach residents how to incorporate integrative medicine into clinical practice, including billing and coding.

## Methods

Study approval was granted by the University of Arizona Institutional Review Board. All participants provided written informed consent.

From 2015 to 2019, Psychiatry PGY-3 and PGY-4 residents, as well as child and adolescent psychiatry fellows at UACOMT, were invited to participate in the IMR-Psychiatry elective. Residents and fellows who elected to participate were allotted time in their training curriculum to complete the elective. To receive a certificate of completion, participants were required to attend 70% of the in-person experiential sessions, complete at least 80% of the online curriculum, and pass the final MK test.

Participant progress in the online curriculum was tracked and attendance was taken at each experiential session. At the completion of the academic year, residents/fellows (*n* = 30) participated in a 1:1 semi-structured, 30-min transcribed interview utilizing open-ended questions with a study personnel who did not have an academic supervisory role. Interview transcripts were analyzed applying principles of grounded theory [13]. Various themes emerged from the feedback data during the initial open coding, while subsequent axial coding identified connections among these, organizing key themes (course value, challenges, and recommendations for improvement) types of engagement in the curriculum (online vs. experiential). A qualitative research expert, not involved in the IMR program, reviewed the analysis to guide the study personnel’s consensus on the axial coding process.

Two pre-post measures were used: a MK test and a self-assessment survey of integrative medicine knowledge. The pre-post MK test consisted of 40 multiple choice questions based on course objectives and content for each of the nine units (Introduction to Integrative Medicine, Neural and Mental Health, Wellbeing in Medicine, Mind–Body Medicine, Nutrition, Dietary Supplements, Complementary Medicine, Clinical Practices, Physical Activity, and Nutrition in Children). The MK test was created by the curriculum authors, changed slightly year-to-year based on updated curriculum content, and the pre- and post-versions were identical. In prior studies of IMR, similar tests have been used to confirm knowledge acquisition [6]. The self-assessment survey was a 35-item, two-part survey that assessed self-rated confidence on a 5-point Likert scale (Strongly Disagree to Strongly Agree) regarding integrative medicine knowledge (eight items) and practice skills (nine items) (Table 1), as well as application to specific medical conditions (18 items) (Table 2).

**Table 1:** Self-assessment of integrative medicine knowledge and skills, based on a 5-point Likert scale (strongly disagree to strongly agree)

Item	*N*	Pre	Post	*p*	Cohen* d*
I can identify authoritative sources about botanicals	31	1.71	3.84	< 0.001	2.15
I know how to respond to patient questions regarding the use of herbs and supplements or where to seek the relevant information	31	2.00	3.84	< 0.001	2.67
I know how to interpret the labels on herbal medicines	31	1.94	3.74	< 0.001	2.28
I know how to recommend botanicals to patients appropriately and safely	31	1.81	3.39	< 0.001	1.78
I know how to advise patients regarding potential interactions between medications and botanicals/supplements or where to seek the relevant information	31	2.13	3.71	< 0.001	1.54
I know the fundamental components of the anti-inflammatory diet	31	2.61	4.1	< 0.001	1.49
I know the different theoretical and philosophical principles of Traditional Chinese Medicine, Ayurvedic Medicine, homeopathy, and Naturopathy	31	2.1	3.58	< 0.001	1.40
I know how to take a spiritual history	31	2.39	3.77	< 0.001	1.64
I know how to respond to patient's questions regarding the practice of mind–body skills (e.g. meditation, guided imagery, biofeedback) or where to seek the relevant information	31	2.9	4.26	< 0.001	1.48
I know what the fundamental tenets of Integrative Medicine are	31	2.74	4.1	< 0.001	1.62
I am aware of the different physical activity recommendations for children and adolescents	31	2.42	3.77	< 0.001	1.29
I know the science of different mind–body techniques such as meditation, mindfulness, guided imagery, and biofeedback	30	2.9	4.17	< 0.001	1.61
I am aware of the different physical activity recommendations for adults	31	3	4.06	< 0.001	0.95
I know how to facilitate a patient's efforts at changing behaviors	31	3.13	4.06	< 0.001	1.05
I know how to assess a patient's readiness to change behavior	30	3.37	4.27	< 0.001	1.19
I can identify the similarities and differences among the manual medicine modalities of massage, physical therapy, osteopathy and chiropractic	31	3.16	4.03	< 0.001	0.98
I can identify areas in my clinical setting that could be enhanced to promote wellbeing and healing	31	3.81	4.29	0.004	0.57

**Table 2:** Self-assessment of integrative medicine skills for medical conditions, based on a 5-point Likert scale (strongly disagree to strongly agree)

I can provide an integrative medicine approach to:	N	Pre	Post	*p*	Cohen* d*
Attention deficit/hyperactivity disorder	31	2.39	3.94	< 0.001	1.56
Premenstrual dysphoric disorder	31	2.35	3.9	< 0.001	1.28
Autism spectrum disorder	30	2.13	3.57	< 0.001	1.26
Bipolar disorders	31	2.1	3.52	< 0.001	1.43
Obesity	31	2.71	4.1	< 0.001	1.36
Obsessive compulsive disorder	31	2.32	3.68	< 0.001	1.16
Psychotic disorders	31	1.87	3.23	< 0.001	1.22
Irritable bowel syndrome	31	2.48	3.84	< 0.001	1.11
Psychosomatic disorders	31	2.55	3.87	< 0.001	1.04
Sleep disorders	31	2.74	4.06	< 0.001	1.16
Depressive disorders	30	3	4.27	< 0.001	1.34
Metabolic syndrome	31	2.45	3.71	< 0.001	1.09
Eating disorders	31	2.13	3.35	< 0.001	1.04
Pain	31	2.71	3.9	< 0.001	1.08
Trauma-related disorders	31	2.65	3.81	< 0.001	1.06
Diabetes	31	2.58	3.61	< 0.001	0.78
Anxiety disorders	31	3.13	4.13	< 0.001	1.04
Substance use disorders	31	2.45	3.45	< 0.001	1.04

Descriptive statistics were conducted on participant demographic and background characteristics. Paired *t*-tests were conducted on the MK test and self-assessment items. Data was analyzed using IBM® SPSS® Statistics (v 27 SPSS).

## Results

Thirty-five of 37 (94.6%) participants completed the elective requirements. These residents and fellows were from the 2015 (*n* = 10; 29%), 2016 (*n* = 5; 14%), 2017 (*n* = 6; 17%), 2018 (*n* = 7; 20%), and 2019 (*n* = 7; 20%) cohorts. Their age range was 27–47 years old, with 54% male (*n* = 19/35) and 46% female (*n* = 16/35). Regarding racial identity, 59% (*n* = 20/34) identified as White, 29% (*n* = 10/34) as Asian, and 3% (*n* = 1/34) as Black or African American. Regarding ethnicity, 12.1% (*n* = 4/33) identified as Hispanic and 87.9% (*n* = 29/33) as non-Hispanic.

Roughly half (*n* = 18/35; 51.4%) were PGY-3; 25.7% (*n* = 9/35) were PGY-4; and 22.8% (*n* = 8/35) were Child and Adolescent Psychiatry Fellows. Almost half (*n* = 16/35; 45.7%) reported previous personal experience using integrative medicine modalities. Just over half had previous medical school coursework in integrative medicine; about a third (*n* = 11/35; 31.4%) had taken an integrative medicine course as part of their required medical school course load and 20% (*n* = 7/35) had completed an elective integrative medicine course in medical school.

The MK test was completed by 30 participants (*n* = 35; 86%) and demonstrated an increase from 64.7% correct at pretest to 81.6% at posttest (*t*(29) =  − 11.1; *p* < 0.001). Thirty-one participants (*n* = 35; 89%) completed the pre-post self-assessment instrument, with each of the 35 integrative medicine knowledge and skill items increasing significantly (*p* ≤ 0.004). Items with the greatest pre-post change were the following: identify authoritative sources about botanicals; know how to respond to patient’s questions regarding the use of herbs and supplements or where to seek the relevant information; and interpret the labels on herbal medicines (Table 1). Items concerning confidence in providing an integrative medicine approach to 18 common medical conditions all increased significantly pre to post (*p* < 0.001). The conditions with the greatest change were the following: attention deficit/hyperactivity disorder; premenstrual dysphoric disorder; autism spectrum disorder; and bipolar disorders (Table 2).

From the post-course semi-structured interviews, initial open coding identified several themes in participants’ course feedback including types of engagement with curriculum, motivation, advantages and disadvantages of various course components, course value, and recommendations for improvement. During axial coding, overlapping themes were distilled into three key themes: course enjoyment, challenges of participation, and recommendations for improvement. Given that each of these was specific to *types of engagement*, we analyzed these themes as they related to each of the major categories: online vs. experiential.

All participants highly recommended the course to other residents and fellows. While time commitment was a generalized challenge for course participation, it was outweighed by the overall value of the course. Each course component presented unique opportunities for its value, challenges, and related recommendations for improvement.

The value of the online component was characterized as structured with clear learning objectives, evidence-based, and useful. Challenges specific to the online component included the quantity and details of online materials being overwhelming and necessitating “cherry picking.” Recommendations for improvement included making online materials permanently accessible to diminish the urgency of reading as much as possible within course time constraints, condensing content to optimize course efficiency, and summarizing key points for each online module.

Participants characterized the value of the experiential component as contributing to participant wellness (such as nutrition, diet, and exercise being “no brainers” and easy to implement, and sleep applications were very helpful), and being more engaging through hands-on learning. One challenge noted from the experiential component was lack of sufficient time for all participants to experience all approaches (i.e., biofeedback). Related recommendation for improvement of this component was to add Tai Chi, acupuncture, cranio-sacral therapy, and Reiki.

## Discussion

The modest yet significant gain in medical knowledge between pre- and post-intervention scores, despite a small sample size, suggests the curriculum increases baseline integrative medicine knowledge; additionally, benefit was seen across multiple domains including self-rated confidence in treating various disorders from an integrative medicine lens, and qualitative reports of applying integrative medicine knowledge for participants’ own health and well-being.

It is essential for psychiatrists to receive training in evaluating the appropriateness and potential risks of various CAM approaches to ensure optimal patient care and avoid potential harm (e.g., medication-supplement interactions and unnecessary costs). Furthermore, despite the expanding research on the therapeutic benefits of diet in psychiatric illnesses, there is a curricular gap in psychiatry residency training regarding nutritional interventions [14]. Similarly, although there is a literature base to support the use of mindfulness in psychiatry residency education to reduce burnout and improve patient care, many residency programs lack sufficient content in this area [10]. By equipping psychiatric residents and fellows with an academic foundation in integrative medicine, IMR-Psychiatry prepares them to make informed, evidence-based decisions when incorporating integrative approaches into patient care. This knowledge expansion has the potential to positively influence clinical decision-making and ultimately contribute to improved patient outcomes [15, 16].

Recorded interviews of participants upon completion of the program provided valuable insights into their experiences with IMR-Psychiatry. Their emphasis on the feasibility of completing the program during their training and acknowledgement of the relevance of integrative medicine in contemporary psychiatric practice was underscored by the high rate of completion of the elective. The positive feedback and endorsement by participants of IMR-Psychiatry indicate its potential as an effective educational tool.

While the outcomes of this study are promising, it is important to acknowledge limitations. The sample size was relatively small and from a single institution, with participants who elected to complete the curriculum, and notably no control group. Future studies with larger, more diverse samples from multiple institutions would be valuable to validate these results and assess the generalizability of IMR-Psychiatry’s effectiveness across various training programs and settings. Given a large portion of the results were based on resident self-assessment, future studies need to include additional measures of resident competency such as evaluation by faculty supervisors. Although the core content of the online curriculum remained largely unchanged, it was updated annually to reflect advancements in medical science. Additionally, IMR-Psychiatry underwent iterative changes based on feedback across the 5-year period examined, resulting in slight variations in the experiential component.

In conclusion, this study highlights the feasibility and other positive early results of the IMR-Psychiatry curriculum in enhancing participants’ MK and confidence in applying integrative medicine to common psychiatric conditions. These findings suggest the curriculum contributes to preparing trainees to provide a multi-faceted and person-centered approach.

Enhancements to psychiatric practice through integrative medicine can deepen whole person and culturally congruent care in a way that simultaneously supports provider well-being. These data pave the way to further develop and expand the IMR-Psychiatry curriculum, including the provision of integrative medicine training to more trainees at residency programs nationally and implementing a multi-site study of the curriculum.

## Data Availability

The datasets generated and analyzed during the current study are available from the corresponding author on reasonable request.
